# Inversion of the physical properties of seafloor surface sediments based on AUV sub-bottom profile data in the northern slope of the South China Sea

**DOI:** 10.1038/s41598-021-86161-x

**Published:** 2021-03-22

**Authors:** Qingjie Zhou, Xishuang Li, Bigui Huang, Lejun Liu, Shan Gao, Hang Zhou, Jie Liu, Baohua Liu, Chengyi Zhang

**Affiliations:** 1 Key Laboratory of Marine Geology and Metallogeny, First Institute of Oceanography, MNR, Qingdao, 266061 People’s Republic of China; 2 Function Laboratory for Marine Geology, Pilot National Laboratory for Marine Science and Technology, Qingdao, 266061 People’s Republic of China; 3 National Deep Sea Center, State Ocean Administration, Qingdao, 266237 People’s Republic of China; 4 CNOOC Research Institute, Beijing, 100027 People’s Republic of China

**Keywords:** Ocean sciences, Physical oceanography

## Abstract

Based on the seafloor reflection coefficient obtained from autonomous underwater vehicle (AUV) sub-bottom profile survey data of the northern slope of the South China Sea, combined with the sample test data of seafloor surface sediments, we use the Biot–Stoll model to establish the equations relating the seafloor reflection coefficient to the porosity, density, and mean grain size of the sediments at the dominant frequency of 5 kHz (the dominant frequency of the AUV sub-bottom profiler). The physical property parameters such as the porosity, density, and mean grain size of seafloor surface sediments are further inverted. Comparison of inversion results with measured results shows that the overall deviation ratios of the inverted mean grain size, porosity, and density of the surface sediments are in the ranges of − 13.56 to 14.44%, − 6.15 to 8.06%, and − 10.85 to 0.46%, respectively. Among them, the mean grain size directly reflects the size of seafloor sediment particles, and the particles are finer in deeper water. Overall, the inversion results are basically consistent with the measured values and thus can well reflect the variation characteristics of the physical properties of seafloor surface sediments.

## Introduction

Sub-bottom profiling is a simple, rapid, and low-cost acoustic method for surveying the structure of seafloor shallow geological strata. Its acoustic signal contains information on the elastic parameters of the subsurface medium and is also rich in geological information^1^. At the seabed (the interface between water and sediments), the sub-bottom profile usually corresponds to the strong reflection of the first positive polarity, with an intensity depending on the acoustic impedance and the acoustic reflection coefficient at the seabed^2–5^. The reflection coefficient and acoustic impedance are closely related to physical properties such as the density, P-wave velocity, water content, porosity, and grain size of the sediments^6–8^. These acoustic and physical properties of seafloor sediments are studied mainly by methods such as sampling or in situ measurements, empirical equation prediction, and theoretical model prediction. At present, acoustic propagation theories of seafloor sediments mainly include fluid theory, elastic theory and porous elastic theory, and the models for acoustic propagation mainly include the Wood equation^9^, the Gassmann equation^10^, the Buckingham model^11,12^ and the Biot–Stoll model^13–17^. Among them, Wood's equation ignores the force generated by the contact between sediment particles, and does not consider the relative movement between pore fluid and skeleton, so it has a large prediction error of sound velocity and application limitations. Based on the elastic theory, the Gassmann equation takes into account the fluid saturation and the elastic properties of solid particle skeleton, but ignores the relative motion of pore fluid and medium skeleton. The Buckingham model assumes that sediment particles are in contact with each other but not cemented, and that there is a viscous force between the particles, acting as both a fluid and an elastic solid^18,19^. As a theory of porous elasticity, Biot–Stoll model takes both porosity and elasticity into account, which can better describe the propagation characteristics of elastic waves under the interaction of solid and fluid stress. In particular, the Biot–Stoll model is highly accurate and widely used to predict the acoustic parameters of sandy sediments^20^. In addition, the use of the Biot–Stoll model and sub-bottom profile data to invert the physical properties of seafloor surface sediments is becoming an emerging direction for the application of sub-bottom profile data. Chirp sub-bottom profile data have been used successfully to quantify the physical property parameters of seafloor surface sediments. For instance, Schock (2004) used Chirp sonar data and the Biot–Stoll model to invert the physical properties (e.g., velocity, density, porosity) of the sediments of Fort Walton Beach in the United States and the seafloor of the South China Sea, and the inversion results strongly agreed with the laboratory measurements^21,22^. Based on Biot’s theory and equivalent fluid density model, Chiu et al. (2015) treated the sub-bottom profile of North Mien-Hua Canyon in northeastern Taiwan Island based on the Biot theory and an equivalent fluid density model to invert the sound velocity, density, and attenuation gradient of seafloor surface sediments, improving the accuracy of the inversion results to some extent^23^. Chen et al. (2017) inverted the physical properties (porosity, density) of seafloor sediments in the Qiongzhou Strait based on the Biot–Stoll model and the Chirp sub-bottom profile data and introduced the Gardner empirical formula to supplement the physical property inversion of the sediments in the high-reflection zone (seafloor reflection coefficient > 0.45); the inversion results were slightly different from the measured values but agreed overall^24^.

As autonomous underwater devices such as autonomous underwater vehicles (AUVs) have matured, AUV-based submarine bathymetric topography and seafloor sub-bottom profiling have become a current research focus as well as a future developmental trend^25–27^. AUVs can be operated at any depth in the range of their operating water depth and are less susceptible to harsh sea surface weather and marine environments in the working process. The AUV data have less interference from ambient noise and higher resolution than the data acquired by shipboard sub-bottom profiling. Theoretically, the physical properties of seafloor surface sediments inverted from AUV sub-bottom profile data are relatively highly reliable. Therefore, in this study, based on the AUV sub-bottom profile survey data of the northern slope area of the South China Sea as well as the seafloor surface sediment sampling and testing data of this area, the correlation between the seafloor reflection coefficient and the physical properties of the sediments is established using the Biot–Stoll model. The physical properties (porosity, density, mean grain size) of the seafloor surface sediments are inverted from the seafloor reflection coefficients that are calculated using the sub-bottom profile data, then compared with the measured physical properties at the sampling points to evaluate the applicability of this method. Our paper provides a new reference method for the rapid acquisition of continuous physical properties of seafloor sediments.

## Study area overview and data sources

The northern slope of the South China Sea is topographically complex and has developed a series of submarine canyons^28,29^. It has a water depth of 400–2500 m. There are two genetic types of seafloor surface sediments in this area^30^. One is the modern fine-grained clastic substance transported by rivers, and the other is the residual sediments that were at low sea level during the early Late Pleistocene Ice Age. Influenced by the characteristics of the sediments themselves in the source area and the effect of the late modification, the grain components of the sediments are characterized by staggered deposition of coarse- and fine-grained sediments, and the high content of coarse-grained materials is mainly a product of long-term deposition in high-energy environments and could have been covered by fine-grained sediments during the sea level rise of the late Ice Age^31^. The seafloor sediments in the northern slope of the South China Sea exhibit distinct zonal distribution characteristics^32^; the sediments in the shallow-water area are dominated by silty clay and silty sand, while sediments in the deep-water area (deeper than 1000 m) are mainly composed of fine-grained clay silt and silty clay^33,34^. On the northern slope of the South China Sea, the water depth varies considerably, the sedimentation rate is high^35,36^. The turbidity currents and mass transport are strong in this area^37,38^. The porosity of the sediments varies over a wide range. Therefore, the distribution of acoustical physical properties of sediments varies obviously^39^.

**Figure 1:**
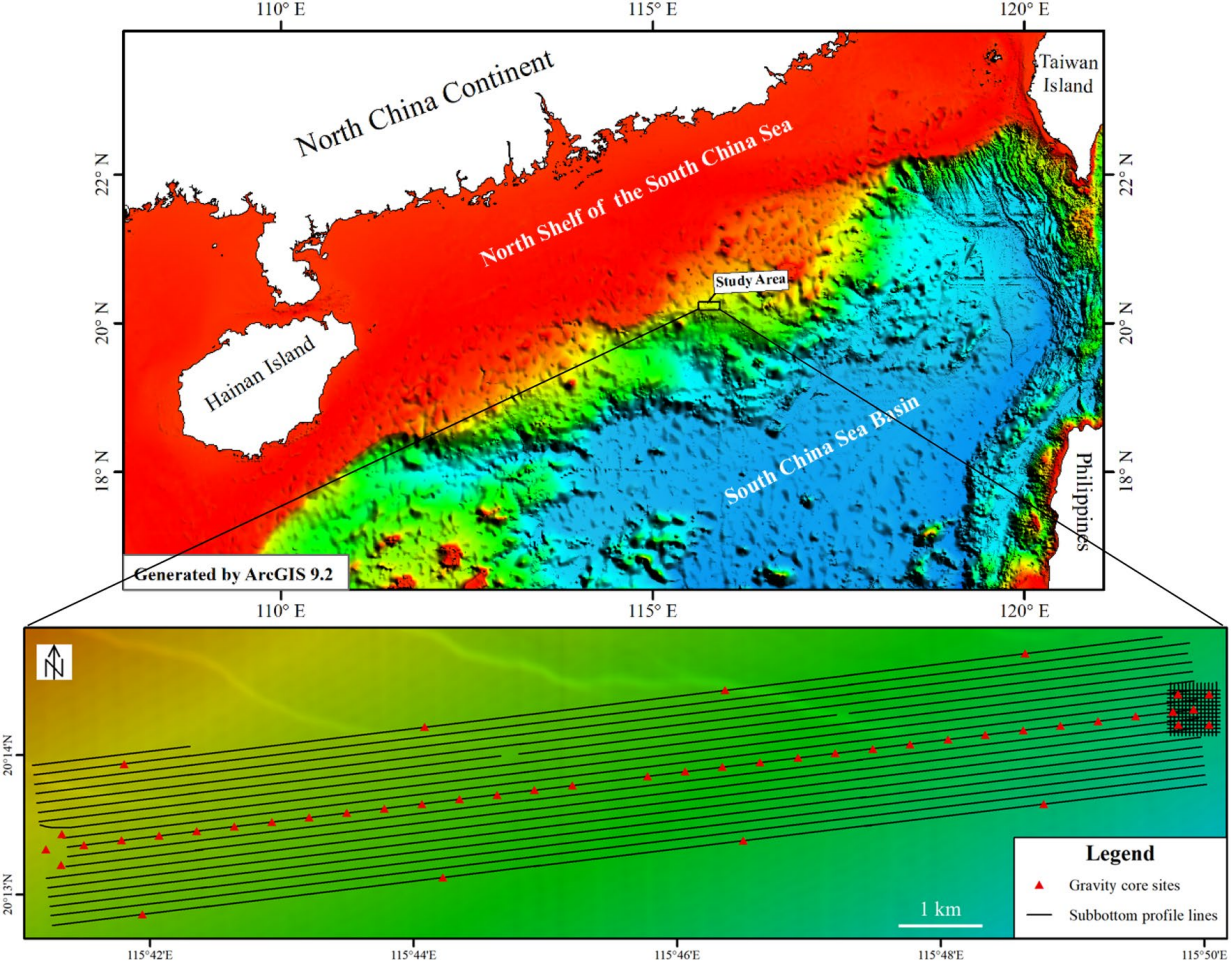
Location of the study area and distribution of survey lines and sampling stations (Generated by ArcGIS 9.2, Data source: Global seabed topography GECBO 15″ × 15″ grid data, http://www.gebco.net): the sub-bottom profile lines are 270 km, with 45 sampling stations.

Figure 1 shows the location of the study area and the distribution of sub-bottom profile survey lines and sampling stations. There are 18 shallow profile lines in the near east–west direction and twenty-five short profile lines in the eastern small area, with a total of 270 km, and 45 sampling stations. The sub-bottom profile data were acquired using an EdgeTech 2200-M sub-bottom profiler onboard a COSL Explorer AUV, with an operating frequency range of 2–16 kHz. Used for deep-sea marine engineering surveys, the COSL Explorer AUV is manufactured by ISE (Canada). It adopts a modular design and has a streamline torpedo structure. It is deployed and recovered through the landslide-type LARS system with a maximum working depth up to 3000 m and can carry out bathymetric surveys, seafloor topographic surveys, and sub-bottom geological surveys in deep waters in accordance with scheduled mission plans.

The core samples of seafloor sediments were taken by the Offshore Oil 708 ship in February 2015 using a deep-water gravity core sampler, with a length of 6 m for each sample. The samples were retrieved, and water content analysis, grain size analysis, and soil specific gravity testing were performed in the laboratory to obtain parameters such as water content, grain size, wet density, void ratio, and grain density.

## Biot–Stoll model and relation establishment

### Biot–Stoll model

The Biot model, as a classical theoretical model for porous elasticity^13^, considers both the porosity and elasticity of a medium and is widely used in the description of anisotropic and viscoelastic two-phase saturated porous media. On the basis of the Biot model, Stoll proposed the Biot–Stoll model^40^, which was applied to calculate sound velocity and attenuation in seafloor sediment medium. This theory holds that solid grains constitute an elastic skeleton that is coupled with pore fluid. Considering the characteristics of the poor cementation between sediment grains and the low modulus of the skeleton, skeleton dissipation is believed to be an important cause of acoustic energy attenuation. Skeleton dissipation is independent of frequency, while fluid viscous dissipation varies with frequency. The 13 parameters involved in the theoretical constitutive equation were determined empirically or by curve fitting. The theoretical elastic modulus H, additional elastic modulus C, and complex elastic modulus M in the Biot model can be expressed as:

1 $$ H = \frac{{(K_{r} - K_{b} )^{2} }}{{D - K_{b} }} + K_{b} + \frac{4}{3}\mu $$

2 $$ C = \frac{{K_{r} \left( {K_{r} - K_{b} } \right)}}{{D - K_{b} }} $$

3 $$ M = \frac{{K_{r}^{2} }}{{D - K_{b} }} $$

4 $$ D = K_{r} \left( {1 + n\left( {\frac{{K_{r} }}{{F_{f} }} - 1} \right)} \right) $$

where $K_{b}$ is the bulk modulus of the skeleton, $\mu$ is the shear modulus of the skeleton, $K_{r}$ is the bulk modulus of the grain, $K_{f}$ is the bulk modulus of the pore fluid, and $n$ is the porosity.

According to the Biot–Stoll model theory, the equation for the propagation of a simple harmonic plane wave in a porous medium can be written as:

5 $$ \left| {\begin{array}{*{20}c} {Hk^{2} - \rho \omega^{2} } \\ {Ck^{2} - \rho_{f} \omega^{2} } \\ \end{array} } \right.\begin{array}{*{20}c} {} & {} \\ \end{array} \left. {\begin{array}{*{20}c} {\rho_{f} \omega^{2} - Ck^{2} } \\ {m\omega^{2} - Mk^{2} - j\frac{\omega F\eta }{\kappa }} \\ \end{array} } \right| = 0 $$

where $\rho$ is the bulk density, $\rho_{f}$ is the pore fluid density, $\omega = 2\pi f$ is the angular frequency, parameter $m = \frac{{c\rho_{f} }}{n}$ is the phase of fluid flow under the macroscopic pressure gradient (where $c$ is tortuosity and n is porosity), *j* is the imaginary unit, $F_{\eta }$ is a viscosity correction factor used to explain the frequency-dependent viscous loss of the oscillatory flow in sediment pores, and $k = \frac{\omega }{v} + j\alpha$ is a complex wavenumber, where the sound velocity $v$ can be calculated as:

6 $$ v = Re\left( {\frac{\omega }{t}} \right) $$

where $Re\left( x \right)$ is the real part of the complex number $x$.

The density of seawater near the seafloor, $\rho_{w}$, is approximately 1025 kg/m^3^, and the sound velocity in seawater, $v_{w}$, is approximately 1530 m/s. The density of seafloor surface sediments can be replaced by the equivalent density^41^:

7 $$ \rho_{eff} = \frac{{\rho \rho^{\prime} - \rho_{f}^{2} }}{{\rho^{\prime} + \rho - 2\rho_{f} }} $$

8 $$ \rho^{\prime} = \frac{{c\rho_{f} }}{n} + \frac{{iF_{\eta } }}{k\omega } $$

Therefore, the seafloor reflection coefficient R can be calculated by the following formula:

9 $$ {\text{R = }}\frac{{{\text{v}}\rho_{{\text{eff - }}} {\text{v}}_{{\text{w}}} {\uprho }_{{\text{w}}} }}{{{\text{v}}\rho_{{\text{eff + }}} {\text{v}}_{{\text{w}}} {\uprho }_{{\text{w}}} }} $$

Biot–Stoll model involves many parameters, and the selection of parameters has different degrees of influence on the calculation results. Parameters such as particle density, porosity, wet density and particle size were obtained through physical property tests. Other parameters, such as permeability, sediment pore factor, particle volume modulus and shear modulus, pore water volume modulus, etc., all need to be calculated by empirical formula or obtained from literature. Detailed parameter values are shown in Table 1.

**Table 1:** Parameters and property interrelationships used to generate inputs for the model.

Input parameter	Parameter value or relationship		References
Grain density $\rho_{{\text{g}}} /({\text{kg/m}}^{3} )$	2700		Measured
Porosity *n*	0.25–0.8		
Tortuosity $\alpha$	$$\alpha = \left\{ {\begin{array}{*{20}c} {1.351.35} & {\quad \varphi \le 4} \\ { - 0.3 + 0.4125\varphi } & {\quad 4 < \varphi < 8} \\ {3.0} & {\quad \varphi \ge 8} \\ \end{array} } \right.$$	$\varphi = - \log_{2} {\text{d}}$, $\varphi$ is the mean grain size, ( unit: $\varphi$), d is the mean grain diameter, (unit: mm)	Schock, 2004^21^
Permeability $\kappa /{\text{m}}^{2}$	$\kappa = \frac{{{\text{d}}^{2} {\text{n}}^{3} }}{{180(1 - {\text{n}})^{2} }}\frac{1}{{\sqrt {10} }}$		Schock, 2004^21^
Absolute Viscosity $\eta /({\text{Pa}} \cdot {\text{s}})$	0.001		Williams, 2002^20^
Grain bulk modulus ${\text{K}}_{{\text{g}}} /{\text{Pa}}$	3.2 × 10^10^		Williams, 2002^20^
Fluid bulk modulus ${\text{K}}_{{\text{w}}} /{\text{Pa}}$	2.395 × 10^9^		Williams, 2002^20^
Fluid density $\rho_{{\text{w}}} /({\text{kg/m}}^{3} )$	1023		Williams, 2002^20^
Frame shear modulus $\mu_{0} /{\text{Pa}}$	$\mu_{0} = 1.835 \times 10^{5} \left( {\frac{n}{1 - n}} \right)^{ - 1.12} \sqrt {\tau_{{\text{a}}} ({\text{z}})}$ $\tau_{{\text{a}}} ({\text{z}}) = (1 - {\text{n}})(\rho_{{\text{s}}} - \rho_{{\text{f}}} ){\text{gz}}$, $\tau_{{\text{a}}} ({\text{z}})$ is the effective stress, gravitational accelerationn $g = 9.8\,{\text{m}} \cdot {\text{s}}^{ - 2}$, z is the depth beneath the sea bed, unit: m		Yamamoto, 1989^42^
Fram bulk modulus ${\text{K}}_{0} /{\text{Pa}}$	${\text{K}}_{0} = \frac{{2\mu_{0} (1 + \sigma )}}{3(1 - 2\sigma )}$ $\sigma$ is the Poisson’s ratio of sediment frame		Yamamoto, 1989^42^
Pore size ${\text{a}}$	${\text{a}} = \frac{{\text{d}}}{3}\frac{{\text{n}}}{{1 - {\text{n}}}}$		Hovem, 1979^43^
Bulk log decrement $\delta_{{\text{f}}}$	$\delta_{{\text{f}}} ({\text{z}}_{{\text{s}}} ) = \delta_{{\text{f}}} ({\text{z}}_{0} )\sqrt {\frac{{{\text{z}}_{0} }}{{{\text{z}}_{{\text{s}}} }}}$		Stoll, 1977^44^

### Relation between seafloor reflection coefficient and physical properties of sediments

**Figure 2:**
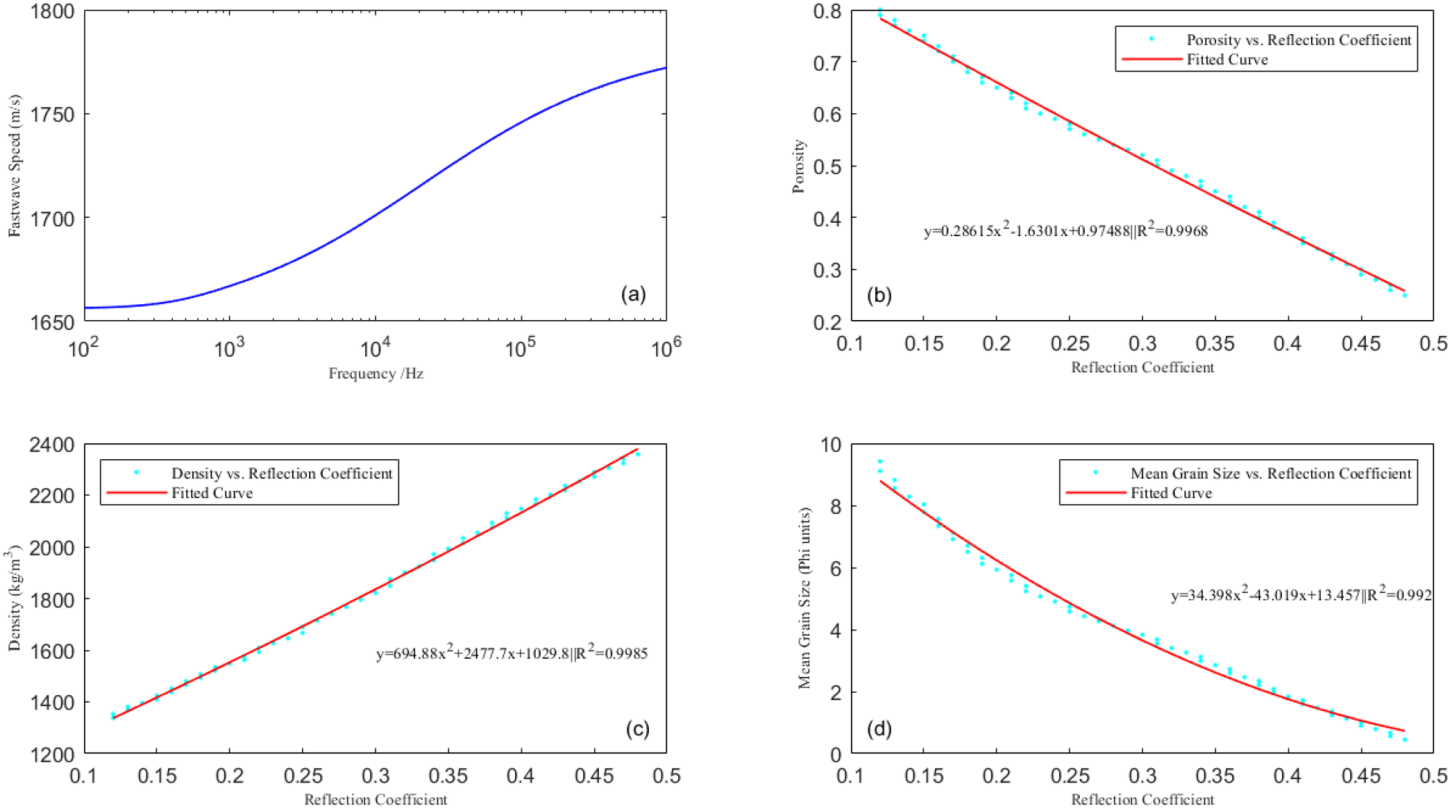
The seafloor sound velocity varies with frequency and the correlations between seafloor reflection coefficient and sediment physical properties: (**a**) variation in sound velocity and frequency; (**b**) variation in reflection coefficient with porosity (f = 5 kHz); (**c**) variation in reflection coefficient with density (f = 5 kHz); (**d**) variation in reflection coefficient with mean grain size (f = 5 kHz).

Based on the Biot–Stoll model and the equation for the equivalent density of sediments, the variation in the seafloor reflection coefficient with frequency is studied; the correlations between the seafloor reflection coefficient and the porosity, density, and mean grain size of the sediments are calculated at a frequency of 5 kHz (the dominant frequency of the Chirp sub-bottom profiler); and fitting equations are established (shown in Fig. 2). Figure 2a shows the variation relationship between the seafloor sound velocity and frequency calculated by the Biot–Stoll model. The sound velocity increases with the increase of frequency, but the speed increases rapidly between 1 and 100 kHz, and then tends to be stable. The frequency band of the sub-bottom profile ranges from 1 to 10 kHz, just in the sound velocity rapidly changing area. Therefore, it is very important to select the appropriate input frequency. The main frequency of the AUV sub-bottom profile data used for inversion in this paper is around 5 kHz, so we choose 5 kHz as the input frequency. Figure 2b shows the variation in the seafloor reflection coefficient with the porosity of the sediments: the reflection coefficient increase with decreasing porosity. To some extent, the change of porosity reflects the change of sediment density, that the higher the porosity, the higher the water content, and the lower the sediment density. The density is directly proportional to the reflection coefficient of the seafloor, and the higher the density is, the greater the reflection coefficient is. As shown in Fig. 2c, it can be seen that the relationship is approximately linear. Figure 2d shows the variation in the seafloor reflection coefficient with the mean grain size of the sediments. It can be seen that the reflection coefficient is negatively correlated with the mean grain size and decreases with increasing mean grain size. It shows that the reflection intensity in the coarse particle area is higher than that in the fine particle area.

## Calculation of the seafloor reflection coefficient

**Figure 3:**
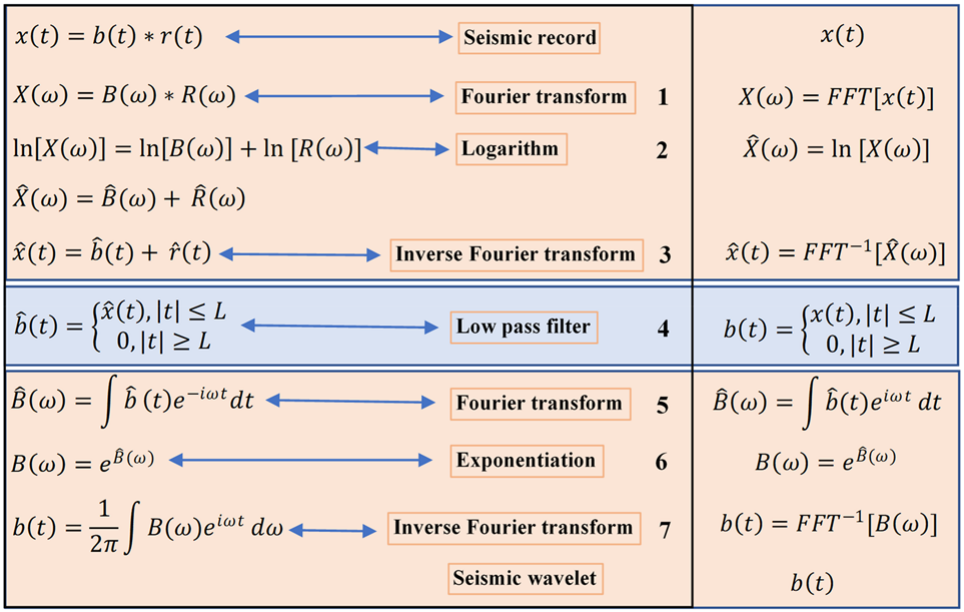
Schematic diagram of the process of using logarithmic decomposition to obtain wavelets.

Seismic records can be regarded as the convolution result of a Gaussian reflection coefficient sequence and a minimum phase seismic wavelet^45^. In the absence of logging data, the statistical method can be used to estimate the wavelet, and the correlation reflection coefficient sequence can be further calculated by using the estimated wavelet^46–48^. Current methods for extracting wavelets mainly include direct observation, autocorrelation (Wold–Kolmogorov method), the Claerbout algorithm, polynomial rooting, logarithmic decomposition (homomorphic filtering method), and the use of logging data to extract wavelets (which is only applicable to well areas). Among these, logarithmic decomposition (homomorphic filtering method) does not require strong assumptions, does not require assumptions for wavelets, does not require us to know the type of wavelets or make the white-noise assumption for the reflection coefficient, and only requires that the logarithmic spectrum of the wavelets be temporally separable from the logarithmic spectral series of the reflection coefficient. Therefore, in this study, logarithmic decomposition (homomorphic filtering method) is used to extract wavelets (as shown in Fig. 3), and then the deconvolution between wavelets and sub-bottom profile data is conducted to obtain the seafloor reflection coefficient.

**Figure 4:**
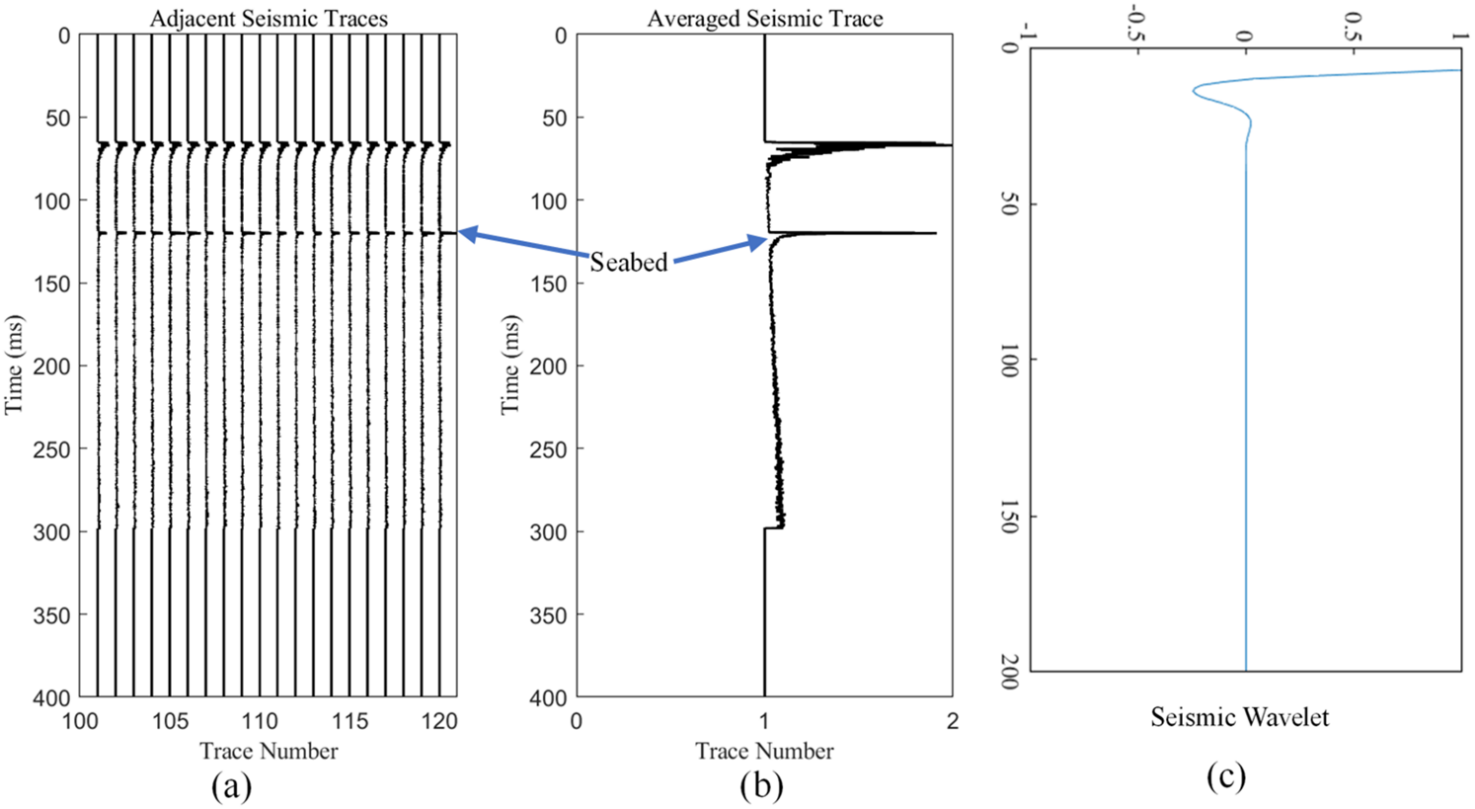
Averaging of the sub-bottom profile track data and the obtained wavelet: (**a**) the extracted adjacent traces with good lineup consistency; (**b**) the averaged seismic trace; (**c**) the wavelet obtained by logarithmic decomposition.

**Figure 5:**
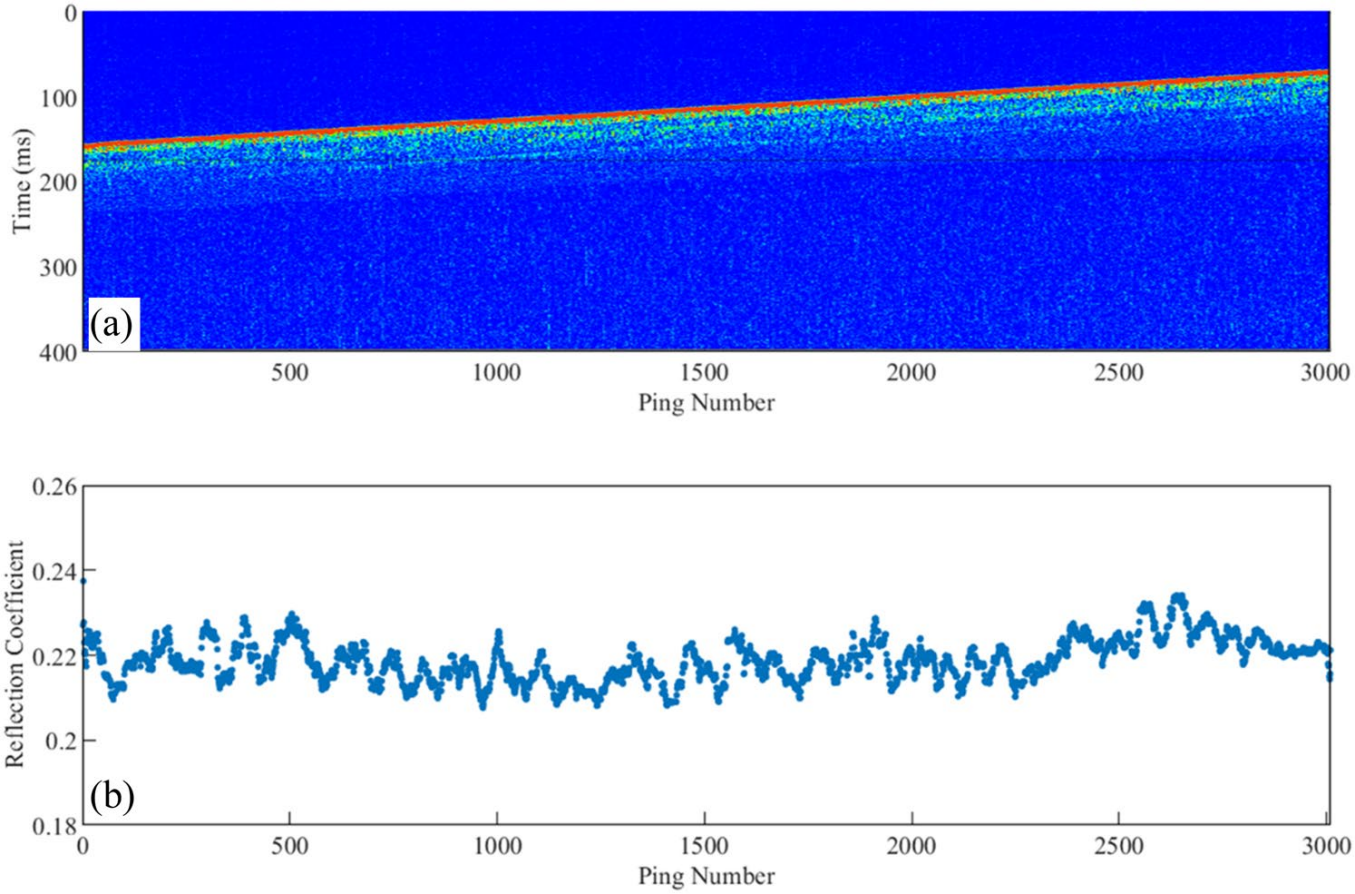
An example subbottom profile and the calculated seafloor reflection coefficient: (**a**) a subbottom profile; (**b**) the calculated seafloor reflection coefficient.

To better obtain the seismic wavelets, the adjacent data with good lineup consistency are selected (Fig. 4a), and their average is calculated to obtain the averaged seismic trace (Fig. 4b), which is then used to extract the wavelets (Fig. 4c). Finally, the convolution between the extracted wavelets and the sub-bottom profile data is carried out to obtain the seafloor reflection coefficient. Figure 5 shows an example of the sub-bottom profile and the obtained seafloor reflection coefficient.

## Comparison of inversion results and measured results

**Figure 6:**
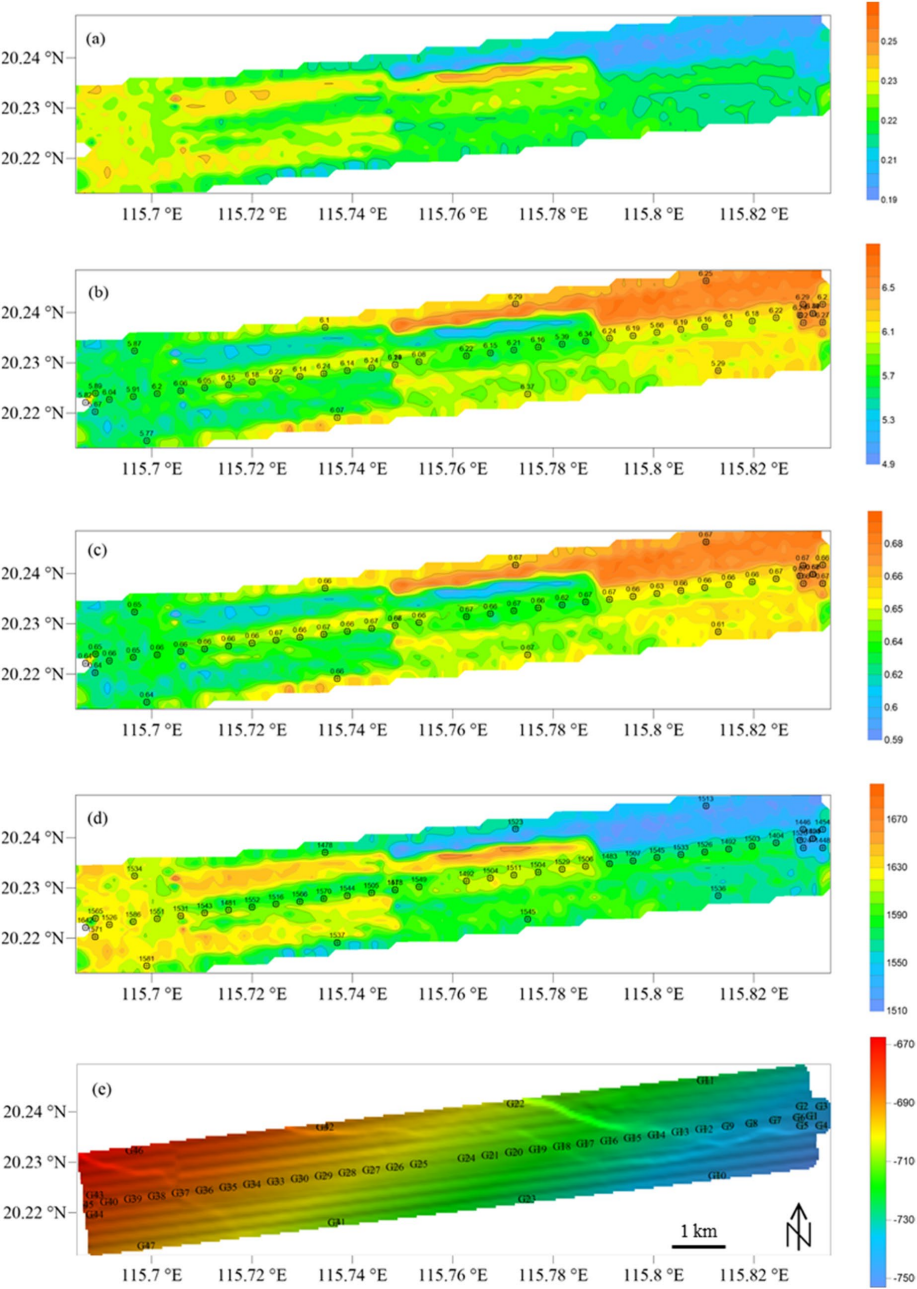
Comparison of the inversion results and sample test results of the physical properties of seafloor surface sediments: (**a**) seafloor reflection coefficient; (**b**) mean grain size (unit:${{ \varphi }}$); (**c**) porosity; (**d**) density (unit: g/m^3^); (**e**) seafloor topography and sampling station.

**Figure 7:**
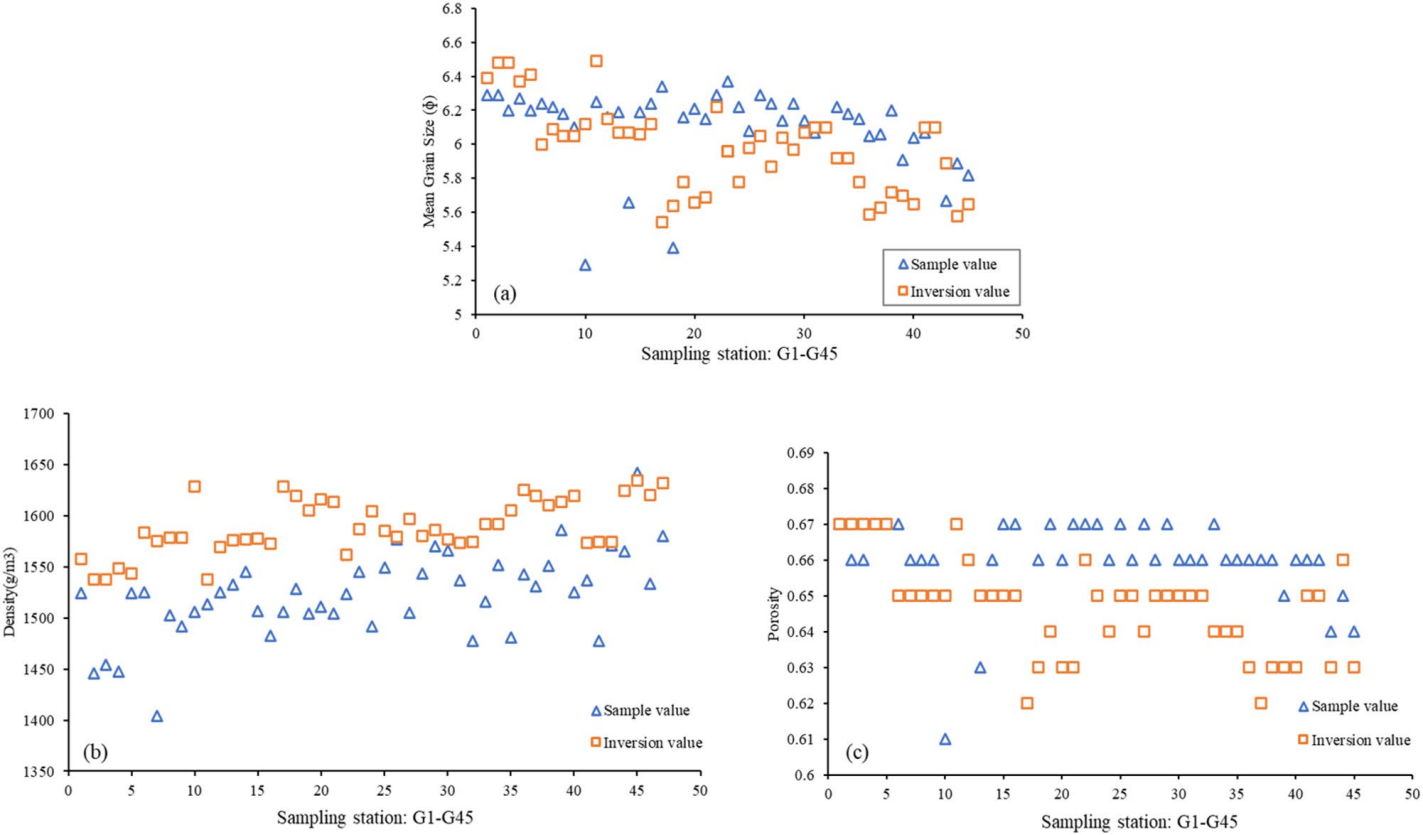
Comparison of inversion results and sample test results at sampling stations (G1–G45): (**a**) Comparison of mean grain size; (**b**) comparison of density; (**c**) comparison of porosity.

**Figure 8:**
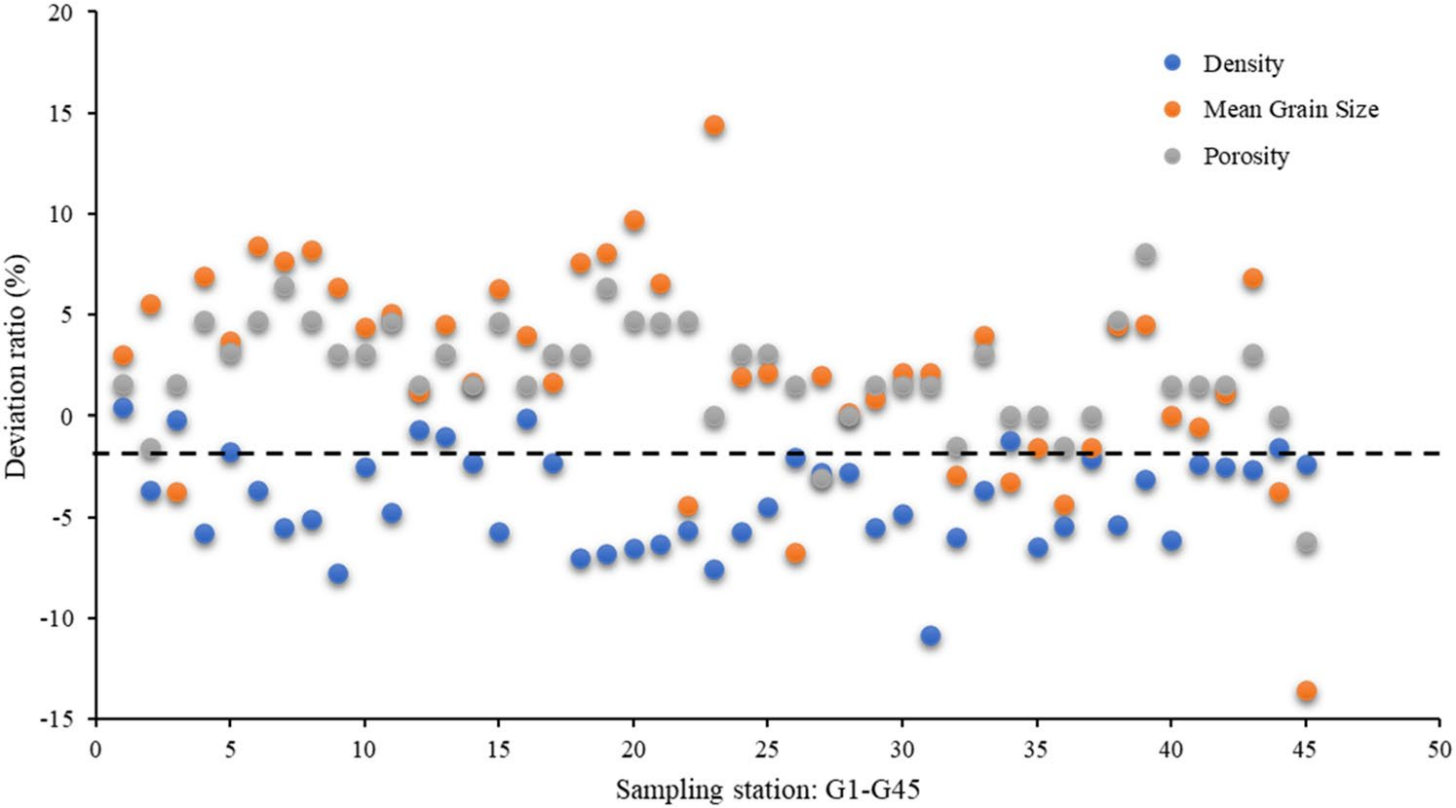
The deviation ratio of inversion results to sampling results.

The sub-bottom profile data in the study area are processed to calculate the seafloor reflection coefficient, which is then used to calculate the physical parameters (mean grain size, porosity, density) of seafloor surface sediments in the study area. The results are shown in Fig. 6. The inversion results of mean grain size near the sampling stations are compared with the sample test results. The mean grain size inversion results are generally larger than the measured values, with a maximum deviation of approximately 0.8 (unit: ɸ, deviation ratio = 14.44%) and an overall deviation ratio in the range of − 13.56% to 14.44%. For the porosity inversion results, the maximum deviation is approximately 0.05 (deviation ratio = 8.06%), and the overall deviation is in the range of − 6.15% to 8.06%. The maximum deviation between the inversion results and the sample test results of the surface sediment density is approximately 170.91 g/m^3^ (deviation ratio = − 10.85%), and the overall deviation ratio is in the range of -10.85% to 0.46%. The inversion results at the sampling station and sample test results are shown in Fig. 7. The uncertainty of inversion results is shown in Fig. 8. Therefore, it is effective to use this method to invert the physical properties of seafloor sediments in this study area. If appropriate model parameters are selected according to the characteristics of the sediment in the study area, this method is also applicable to the whole continental slope area or other areas.

## Discussion

**Figure 9:**
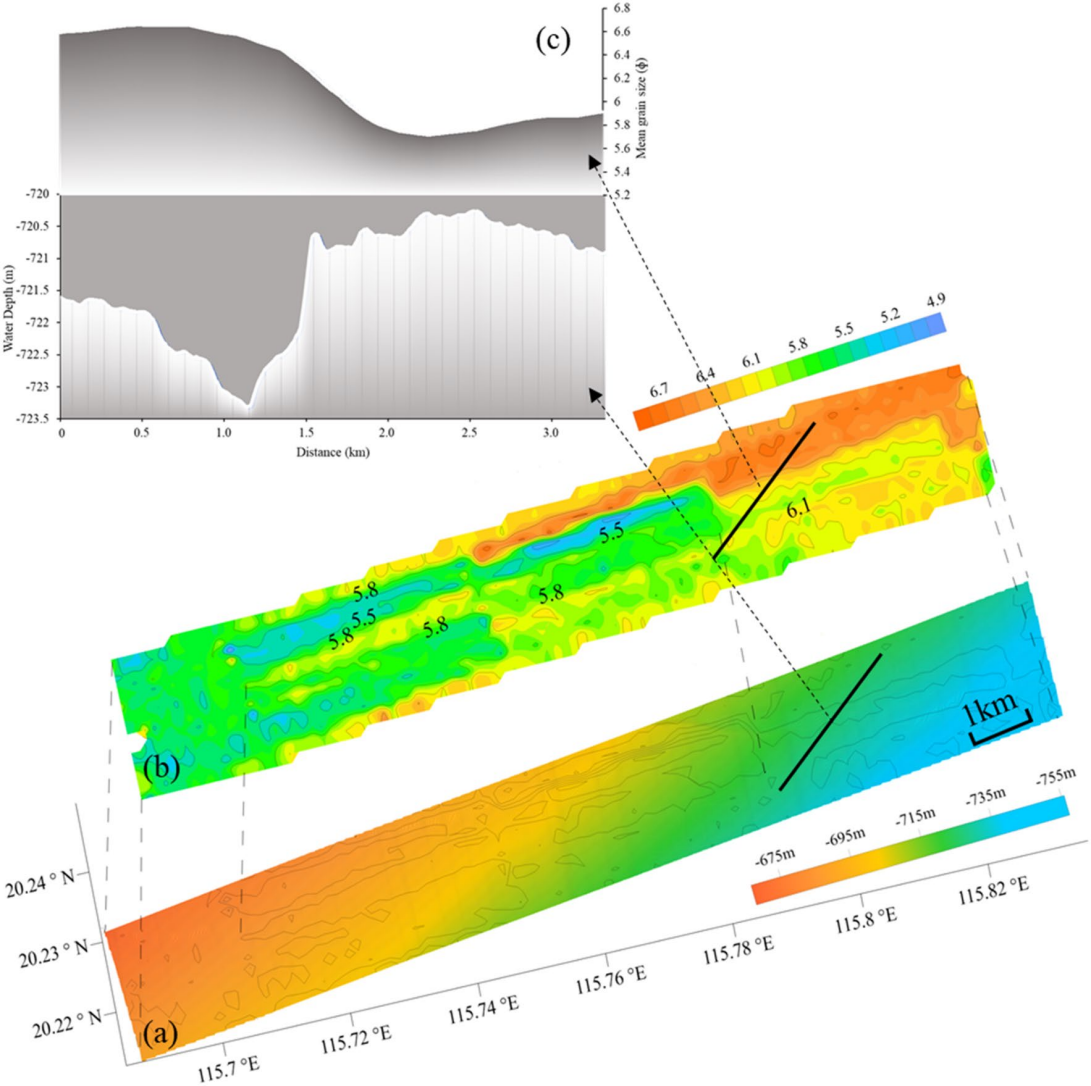
Superimposed contrast diagram of sediment mean grain size and topography: (**a**) is a topographic map superimposed with mean grain size contour; (**b**) is the contour map of mean grain size (unit:${{ \varphi }}$); (**c**) is the intercepted topographic profile and the mean grain size profile at the corresponding position, and the position is the solid black line in (**a**) and (**b**).

The formation of sediment is influenced by provenance, hydrodynamic conditions and topographic and geomorphic factors. However, the seafloor surface sediments in the same study area can show the spatial variation of sedimentary environment through the difference of grain size. In this paper, the scope of the study area is lesser, and relatively flat terrain, water depth changes between 665–760 m. From sampling test and analysis results (the blue triangle in Fig. 7), the mean grain size of the seafloor surface sediment is between 5.29 to 6.37 ${{\varphi }}$, the density between 1404.08 to 1641.84 g/m^3^ and the porosity between 0.61 to 0.67. The sediment types are mainly silty clay and silty sand. It can be seen from the inversion results that the seafloor reflection coefficient decreases with the increase of water depth from west to east in the study area, and the corresponding sediment density decreases gradually, indicating the changing trend of seafloor sediment. The mean grain size directly reflects the size of sediment particles. According to the inversion results, the mean grain size is roughly between 4.9 to 6.8 ${{\varphi }}$. It is basically consistent with the sample test results, and has obvious zoning characteristics. Increases with the increase of the water depth from west to east, of which 4.9–6 ${{\varphi }}$ corresponding medium energy deposition, 6–7 ${{\varphi }}$ corresponding to the medium and low energy with fine grained deposits. Figure 9 can be more intuitive to see the change that the change of the mean grain size and the topography is negative correlation.

In this paper, the inversion of physical properties of seafloor surface sediments based on sub-bottom profile can effectively make up for the gap of discrete sampling and further reflect the variation characteristics of seafloor surface sediments in the study area. However, this method is limited by many factors, such as the quality of sub-bottom profile, the calculation of seafloor reflection coefficient, the selection of model parameters, etc., and its accuracy needs to be further improved.

## Conclusion

Based on the Biot–Stoll model and the AUV sub-bottom profile data of the upper part of the northern slope in the South China Sea, the engineering geological parameters (mean grain size, porosity, density) of the seafloor surface sediments in this area haven been inverted. The following conclusions are drawn:

1. Based on the engineering geological characteristics of the seafloor surface sediments in the study area, the Biot–Stoll model was established. Based on the Biot–Stoll model, the relationship equation between the seafloor reflection coefficient and the porosity, density and average particle size of sediments at the dominant frequency of 5 kHz was calculated. The equations have high goodness-of-fit values, with coefficients of determination (R^2^) all greater than 0.99, providing a reliable basis for the inversion of the physical properties of sediments using the seafloor reflection coefficient.
2. The physical parameters (porosity, density, mean grain size) of seafloor surface sediments have been inverted from the seafloor reflection coefficient that was calculated from the sub-bottom profile data. The inversion results are compared with the sampling test results, and the values of them are close to each other at the sampling points. The overall deviation ratios of the inversion results of mean grain size, porosity, and density of the surface sediments are in the range of − 13.56 to 14.44%, − 6.15 to 8.06%, and − 10.85 to 0.46%, respectively.
3. From the inversion results, with the increase of water depth in the study area from west to east, the seafloor reflection coefficient decreases, the corresponding sediment density decreases gradually, and the mean grain size increases (sediment particle size decreases), indicating the changing trend of seafloor sediment. Compared with discrete sampling points, the inversion results can reflect the changes of seafloor sediment types more intuitively on the surface.
